# Roxadustat (FG-4592) Attenuates Renal Ischemia–Reperfusion Injury but Upregulates the NKG2D Ligand MICA

**DOI:** 10.3390/biom16101437

**Published:** 2026-10-02

**Authors:** Huan Yang, Ningxin Xu, Yong Wu, Ming Cheng, Xiaoyan Qiu, Shaojun Liu, Ming Zhang, Minmin Zhang

**Affiliations:** 1 Department of Nephrology, Huashan Hospital, Fudan University, Shanghai 200040, China; 20211220009@fudan.edu.cn (H.Y.); xu_ningxin@fudan.edu.cn (N.X.); 19211220016@fudan.edu.cn (Y.W.); chengmingedu97@163.com (M.C.); liushaojun@fudan.edu.cn (S.L.); 2 Department of Nephrology, Qilu Hospital (Qingdao), Shandong University, Qingdao 266035, China; 3 Department of Pharmacy, Huashan Hospital, Fudan University, Shanghai 200040, China; xyqiu@fudan.edu.cn; 4 Department of Pharmacy, Tongji Hospital, Tongji University, Shanghai 200065, China

**Keywords:** ischemia–reperfusion injury, kidney transplantation, MICA, NKG2D ligand, Roxadustat

## Abstract

Ischemia–reperfusion injury (IRI) causes acute kidney injury (AKI) and transplant dysfunction. NKG2D ligands (human MICA and murine RAE-1/H60) drive immune activation, while Roxadustat (FG-4592) is reportedly renoprotective. We hypothesized that Roxadustat modulates these ligands in renal IRI. MICA expression was assessed in biopsies from AKI and post-renal transplantation (PRT) patients versus controls. Male Balb/c mice received Roxadustat or vehicle before unilateral IRI, followed by assessment of kidney injury and HIF-1α, RAE-1, and H60 expression. In vitro, human and murine tubular cells underwent hypoxia/reoxygenation with or without FG-4592 pretreatment, and ligand expression was analyzed. Our results showed that MICA expression was elevated in kidney tissues from individuals with AKI or PRT compared with controls. In mice, FG-4592 pretreatment attenuated renal IRI-induced dysfunction and tubular injury, concurrently stabilizing HIF-1α protein levels. Notably, FG-4592 treatment enhanced the expression of murine NKG2D ligands at the transcriptional and translational levels in renal IRI. This effect was corroborated in vitro, where hypoxia/reoxygenation upregulated MICA in human and RAE-1 in mouse tubular cells, a response potentiated by FG-4592. In conclusion, Roxadustat protects against renal IRI but upregulates NKG2D ligands, warranting further study of its mechanisms and consequences in AKI/PRT.

## 1. Introduction

Ischemia–reperfusion injury (IRI) is a key pathophysiological process in acute kidney injury (AKI). While it begins with restricted blood flow, the subsequent reperfusion paradoxically exacerbates tissue damage and triggers profound inflammation [1]. IRI often follows trauma, shock, and organ transplantation [2]. In renal transplantation, it poses an inevitable challenge to allograft outcomes, causing tubular dysfunction that leads to AKI, delayed graft function (DGF), and rejection [3], with consequences including nephron loss, vascular injury, fibrosis, and eventual graft failure [4]. Clinically, IRI contributes to significant morbidity and mortality. Despite numerous attempts to target its potential mechanisms, effective clinical therapies remain elusive [1,3].

Natural Killer Group 2, member D (NKG2D) ligands, including MHC Class I Polypeptide-Related Sequence A (MICA), function as stress-inducible signals. Under normal conditions, their expression on epithelial and endothelial cells is minimal but can be strongly induced by cellular stressors like oxidative stress, tissue injury, inflammation, cancer/cellular transformation, and infection [5,6,7,8]. By binding to NKG2D on immune effector cells like natural killer cells, NKT cells, and γδ T lymphocytes, these ligands activate cytotoxicity and link innate to adaptive immune responses [9]. The MICA gene is absent in rodents. The murine NKG2D ligand family comprises three subfamilies: RAE-1, H60, and mouse UL16-binding protein-like Transcript 1 (MULT-1) [6]. The clinical relevance of NKG2D and its ligands is evident from their links to renal allograft rejection [9] and alterations in ischemic injury responses, including renal IRI [10,11], ischemic lung disease [12], and myocardial infarction [13]. This broad relevance prompts the question of whether the NKG2D/MICA pathway is a feasible therapeutic target in IRI.

The hypoxia-inducible factor (HIF) complex, composed of regulatory α (HIF-1α/2α/3α) and constitutive β subunits, serves as a key transcription factor during IRI. It activates by binding to hypoxia-response elements (HREs), promoting gene transcription involved in ischemic tissue injury and repair [14]. Notably, the HIF-1 variant, comprising HIF-1α and HIF-1β subunits, is a critical transcription factor regulating metabolism in hypoxic kidney injury [14,15]. Given this, HIF stabilization has emerged as a promising therapeutic strategy for IRI. Roxadustat (FG-4592), a clinically used HIF prolyl hydroxylase inhibitor (HIF-PHI), is a highly effective chemical agent that stabilizes HIFs by competitively inhibiting the prolyl hydroxylase domain (PHD). Owing to its ability to induce erythropoietin, it is widely applied for anemia management in chronic kidney disease [16,17]. Preclinical studies, including our prior work [18], have shown that HIF stabilization confers IRI protection through multiple mechanisms, particularly immunomodulation [19,20,21]. Given that both HIF and NKG2D ligands are stress-inducible in IRI, we hypothesized that Roxadustat protects against renal IRI by regulating NKG2D ligands. The current research examined how Roxadustat affects IRI-induced kidney damage and modulates NKG2D ligands (human MICA and murine RAE-1/H60), to provide new perspectives for managing renal IRI.

## 2. Materials and Methods

### 2.1. Human Subjects

Ethical approval for the study involving human tissue samples was obtained from the Ethics Committee of Huashan Hospital, Fudan University (No. KY2016-394 for AKI/PRT biopsy samples and No. KY2022-913 for normal control tissues from nephrectomy specimens), and written informed consent was obtained from all participants. This study enrolled 6 patients with acute kidney injury (AKI) and 6 with post-renal transplantation (PRT) dysfunction, all pathologically diagnosed with acute tubular necrosis (ATN). AKI with concomitant ATN was confirmed by renal biopsy per KDIGO criteria [22]. The AKI cases were community-acquired, and renal biopsy was performed at the time of AKI diagnosis. Based on medical record review and pathological evaluation, the causes were classified as prerenal or intrinsic ischemic injury. Other causes (e.g., immune-mediated diseases or nephrotoxicity) were excluded. In the PRT group, ATN was identified on post-operative biopsy without evidence of allograft rejection. Para-carcinoma tissues from nephrectomies (no AKI history, *n* = 6) served as negative controls. These tissues were obtained from the non-tumor pole of nephrectomy specimens, and a pathologist confirmed that they showed no significant inflammation, tubular atrophy, interstitial fibrosis, or tumor infiltration. Patient information was extracted from the renal biopsy registry and digital medical records of the hospital. Immunohistochemistry and immunofluorescence were employed to detect MICA expression in paraffin-embedded renal biopsy sections, with subsequent microscopic visualization using Leica equipment (Leica Microsystems, Wetzlar, Germany).

### 2.2. Animals

The study procedures involving animals received ethical clearance from the Experimental Research Ethics Committee of Fudan University (Approval No. 2020 Huashan Hospital JS-507). All experimental work was performed at the university’s Laboratory Animal Science facility. Male Balb/c mice (8–10 weeks, SPF) were used. They were fed standard diets and housed under controlled conditions (12 h light/dark cycle, 23–25 °C) with ad libitum access to food and water. A total of 24 mice were used in this study. All 24 mice were included in the in vivo analyses.

### 2.3. Renal IRI Model

Animals were randomly allocated to the experimental groups using randomly selected animal identification numbers. Mice were then allocated into four distinct cohorts (*n* = 6 per group): (1) Sham group subjected to a sham surgical procedure followed by intraperitoneal administration of saline, (2) Sham + FG group receiving FG-4592 (# S1007, Selleck Chemicals, Houston, TX, USA) at 10 mg/kg (prepared at a final concentration of 1 mg/mL in PBS, following initial dissolution in DMSO at 50 mg/mL) 5 days prior to operation, (3) IR group undergoing IR surgery with saline intraperitoneal injection, and (4) IR + FG group treated with FG-4592. Renal IRI was established following described protocols [23]. Animals were anesthetized with pentobarbital sodium (50 mg/kg, i.p.). Following a dorsal incision, the right kidney was excised and a vascular clamp was applied to the left renal artery for 30 min. Throughout the procedure, animals were maintained in a temperature-controlled infant incubator (36–37 °C). Thereafter, the clamps were removed, incisions sutured, and 0.5 mL 0.9% saline was injected intraperitoneally. Control animals underwent unilateral nephrectomy and renal pedicle exposure without ischemia induction. All subjects were euthanized under anesthesia 24 h post-procedure. Blood and left kidney tissues were harvested for subsequent examination. Experimental procedures were performed under standardized conditions. Surgical procedures and intraoperative temperature were kept consistent across groups, and samples were collected at the same predefined time point after reperfusion. Samples from different groups were processed in parallel where feasible.

### 2.4. Cell Culture Procedures

The human renal tubular epithelial HK-2 cell line was kindly provided by Prof. Jing Chen (Department of Nephrology, Huashan Hospital, Fudan University, Shanghai, China). This cell line was originally purchased from the Cell Bank of the Chinese Academy of Sciences and has been described previously [24]. HK-2 cells were cultured in Dulbecco’s Modified Eagle’s Medium/Nutrient Mixture F-12 (DMEM/F12, Gibco, Thermo Fisher Scientific, Waltham, MA, USA) supplemented with 10% fetal bovine serum (FBS, Sigma-Aldrich, St. Louis, MO, USA). The mouse proximal tubular epithelial cells (mTECs) were generously supplied by Dr. Sujuan Xu (Department of Nephrology, The Third Hospital of Hebei Medical University, Shijiazhuang, China) and have been described previously [25]. Similarly, mTECs were grown in DMEM (Gibco, Thermo Fisher Scientific, Waltham, MA, USA) containing 8% FBS (Sigma-Aldrich, St. Louis, MO, USA). Both cell lines were maintained in medium supplemented with 1% penicillin–streptomycin (Beyotime Biotechnology, Shanghai, China) under standard conditions (37 °C, 5% CO_2_ in a humidified atmosphere). All in vitro experiments were performed in three independent experiments, each with duplicate or triplicate wells. For statistical analysis, the independent experiment was used as the unit of analysis (*n* = 3).

### 2.5. Cell Hypoxia/Reoxygenation (H/R) Injury

HK-2 cells along with mTECs were seeded in 6- or 12-well culture plates (containing or lacking coverslips) and maintained under normal conditions for 24 h prior to hypoxic exposure. For the group receiving FG-4592, cells were pretreated with 10 μM of this compound under normoxic conditions for 24 h before hypoxia. The H/R cellular model was created following established protocols from reference [19]. To induce hypoxia, the culture medium was replaced with serum-free solution before a 24 h incubation in an anaerobic chamber (Anaero Pack, Mitsubishi Gas Chemical Co. Inc., Tokyo, Japan). Reoxygenation was then performed for 2 h at 37 °C in a 5% CO_2_/95% air environment. Cells not subjected to H/R constituted the control group. Subsequently, cells were harvested for immunofluorescence staining and protein analysis via Western blot.

### 2.6. Assessment of Renal Function Parameters

Blood specimens underwent centrifugation (3000 rpm, 4 °C, 15 min) to obtain serum. Levels of serum creatinine (SCr) and blood urea nitrogen (BUN) were quantified with an automated biochemical analyzer (Roche c702, KingMed Diagnostics, Shanghai, China).

### 2.7. Histology Examination

Following fixation in 4% paraformaldehyde for 48 h, tissue samples were dehydrated, paraffin-embedded, and sectioned into 3 μm slices for staining with either hematoxylin–eosin (H&E) or periodic acid–Schiff (PAS). Kidney injury scores were assessed in the 6 randomly selected 200× fields, with criteria: 0 (no injury), 1 (<25%), 2 (26–50%), 3 (51–75%), 4 (>75%). For immunohistochemistry and immunofluorescence quantification, six randomly selected cortical fields per slide were analyzed at ×200 or ×400 magnification using ImageJ (version 1.53, NIH, Bethesda, MD, USA), and scoring was performed by two independent investigators blinded to group allocation.

### 2.8. Immunohistochemical Staining

Renal tissues were processed for immunohistochemical examination following established protocols [23]. Dewaxed sections were blocked with 3% H_2_O_2_ (15 min) and 5% BSA (37 °C, 1 h). Tissue sections were subjected to overnight incubation at 4 °C with primary antibodies (anti-MICA [ab62540, Abcam, Cambridge, UK], anti-HIF-1α [NB100-105, NOVUS Biologicals, Centennial, CO, USA], anti-RAE-1 [AF1136, R&D Systems, Minneapolis, MN, USA], and anti-H60 [MAB1155, R&D Systems, Minneapolis, MN, USA]) diluted 1:200. After incubation with biotin-conjugated goat anti-rabbit and anti-mouse IgG for 1 h, signals were visualized with diaminobenzidine (DAB) substrate (GK500705, Gene Tech, Shanghai, China). Sections were then counterstained with hematoxylin and imaged under a Leica microscope (Leica Microsystems, Wetzlar, Germany).

### 2.9. Immunofluorescence Staining

For immunofluorescence, dewaxed sections or cell-climbing slices were exposed overnight at 4 °C to the following primary antibodies: anti-MICA (ab62540, Abcam, Cambridge, UK) and anti-lotus tetragonolobus lectin (LTL, B-1325, Vector Laboratories, Newark, CA, USA). Fluorescein isothiocyanate (FITC)-conjugated secondary antibodies were used. Nuclei were visualized by staining sections with 4′, 6-diamidino-2-phenylindole (DAPI), with imaging performed on a Leica microscope system (Leica Microsystems, Wetzlar, Germany).

### 2.10. Western Blot Analysis

Following homogenization of renal tissues in RIPA Buffer (Beyotime Biotechnology, Shanghai, China), protein concentration was determined using a BCA Protein Assay Kit (Beyotime Biotechnology, Shanghai, China). Western blots used primary antibodies: anti-MICA (ab62540, Abcam, Cambridge, UK), anti-HIF-1α (AF1009, Affinity Biosciences, Cincinnati, OH, USA), anti-RAE-1 (AF1136, R&D system, Minneapolis, MN, USA), anti-H60 (MAB1155, R&D system, Minneapolis, MN, USA). GAPDH (ab181602, Abcam, Cambridge, UK) served as intrinsic quality control. Secondary antibodies included goat anti-rabbit and goat anti-mouse IgG (LI-COR Biosciences, Lincoln, NE, USA; 926-68071 and 926-6870). Protein bands were subsequently visualized using an Odyssey Fc Imaging System (LI-COR Bioscience, Lincoln, NE, USA). Densitometric analysis was performed with ImageJ software (version 1.53, NIH, USA). For each protein, at least three independent biological replicates were analyzed, with duplicate technical runs. Densitometric quantification was based on biological replicates (*n* = 3 or *n* = 6 as indicated in the figure legends), not on technical replicates. Representative blots are shown from one experiment. Full uncropped Western blot images have been uploaded as Supplementary Material.

### 2.11. Real-Time PCR

Total RNA was extracted from mouse renal tissue using Trizol reagent (Thermo Fisher Scientific, Waltham, MA, USA), then reverse-transcribed into cDNA with the HyperScript III 1st Strand cDNA Synthesis Kit (R202, EnzyArtisan, Shanghai, China). Quantitative real-time PCR was subsequently performed with Universal SYBR qPCR Mix (R204, EnzyArtisan, Shanghai, China), using primers listed in Table 1. For RT-qPCR, *n* = 3 mice per group were randomly selected from the 6 mice in each group, and each sample was run in triplicate. Relative expression levels were calculated using the 2^−ΔΔCt^ method, with β-actin as the internal control.

### 2.12. Blinding

The surgeon performing the renal IRI procedure and the investigator administering FG-4592 or saline were aware of the group allocation, as blinding was not feasible for these interventions. However, all outcome assessments were performed in a blinded manner. Specifically, histological injury scoring, immunohistochemical and immunofluorescence image analysis, Western blot densitometry, and qPCR data analysis were conducted by investigators who were blinded to the experimental group assignments. Data were coded and unblinded only after the completion of all analyses.

### 2.13. Statistical Analysis

Data are expressed as mean ± SEM. Statistical analyses were conducted using SPSS 23.0 (IBM Corp., Armonk, NY, USA) and GraphPad Prism 8.0 (GraphPad Software, San Diego, CA, USA). Multi-group comparisons were evaluated by one-way ANOVA and Newman–Keuls post hoc test. All tests were two-sided. *p*-values < 0.05 were defined as statistically significant.

## 3. Results

### 3.1. MICA Expression Was Increased in the Renal Biopsy of AKI and PRT Patients

**Figure 1:**
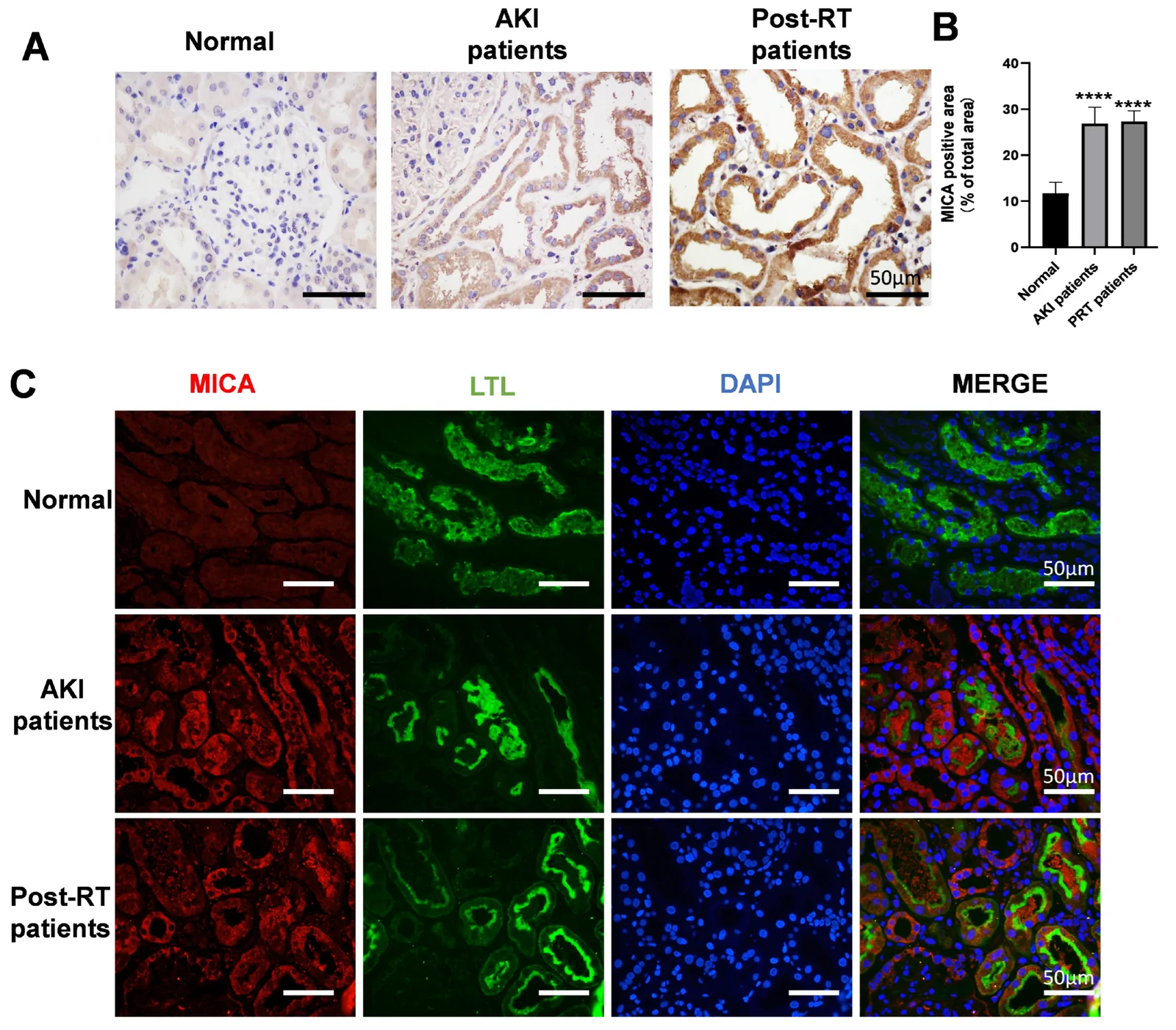
Enhanced expression and tissue distribution of the human NKG2D receptor ligand MICA in renal biopsy of AKI and PRT patients. (**A**) Representative immunohistochemistry staining (×400, bar = 50 μm) of MICA in the renal biopsies from AKI (*n* = 6) and post-renal transplantation with renal insufficient (*n* = 6) patients. (**B**) The semiquantitative analysis of MICA protein levels presented in graphical form. (**C**) Representative immunofluorescence microscopy images (×400 magnification, bar = 50 μm) illustrating co-localization of MICA with LTL, a specific marker for proximal tubular epithelium, in renal biopsies from patient groups compared with controls (*n* = 6). Fluorescent signals are displayed separately for MICA (red), LTL (green), DAPI (blue), and the merged view. Data are expressed as mean ± SEM (*n* = 6). **** *p* < 0.0001 versus the control group. Abbreviations: AKI, acute kidney injury; PRT, post-renal transplantation; LTL, lotus tetragonolobus lectin.

To examine the expression and localization of MICA in renal tissues, we enrolled six AKI and six PRT patients with renal dysfunction, from whom renal biopsy tissue blocks were available for analysis. Patient baseline characteristics are summarized in Table 2. Immunohistochemical staining revealed a marked elevation of MICA expression in patient kidney tissues relative to normal controls (Figure 1A,B; MICA-positive area: normal controls, 11.72 ± 0.98% of total area; AKI, 26.90 ± 1.43%; PRT, 27.32 ± 0.94%; overall ANOVA *p* < 0.0001; both *p* < 0.0001 versus controls). Furthermore, the double-staining with LTL, a proximal tubule marker, indicated the distribution of MICA (Figure 1C). Together, these findings indicated NKG2D pathway activation in renal ATN, whether in the setting of AKI or PRT. These findings indicate NKG2D pathway activation in human renal ATN. Because these patients did not receive Roxadustat, these data support the clinical relevance of MICA upregulation in IRI-associated tubular injury but do not directly assess the pharmacological effect of HIF-PHI treatment, which was investigated in the subsequent murine IRI and in vitro H/R experiments.

### 3.2. FG-4592 Pretreatment-Mitigated Kidney Injury and Stabilized HIF-1α in IR-AKI Murine Model

**Figure 2:**
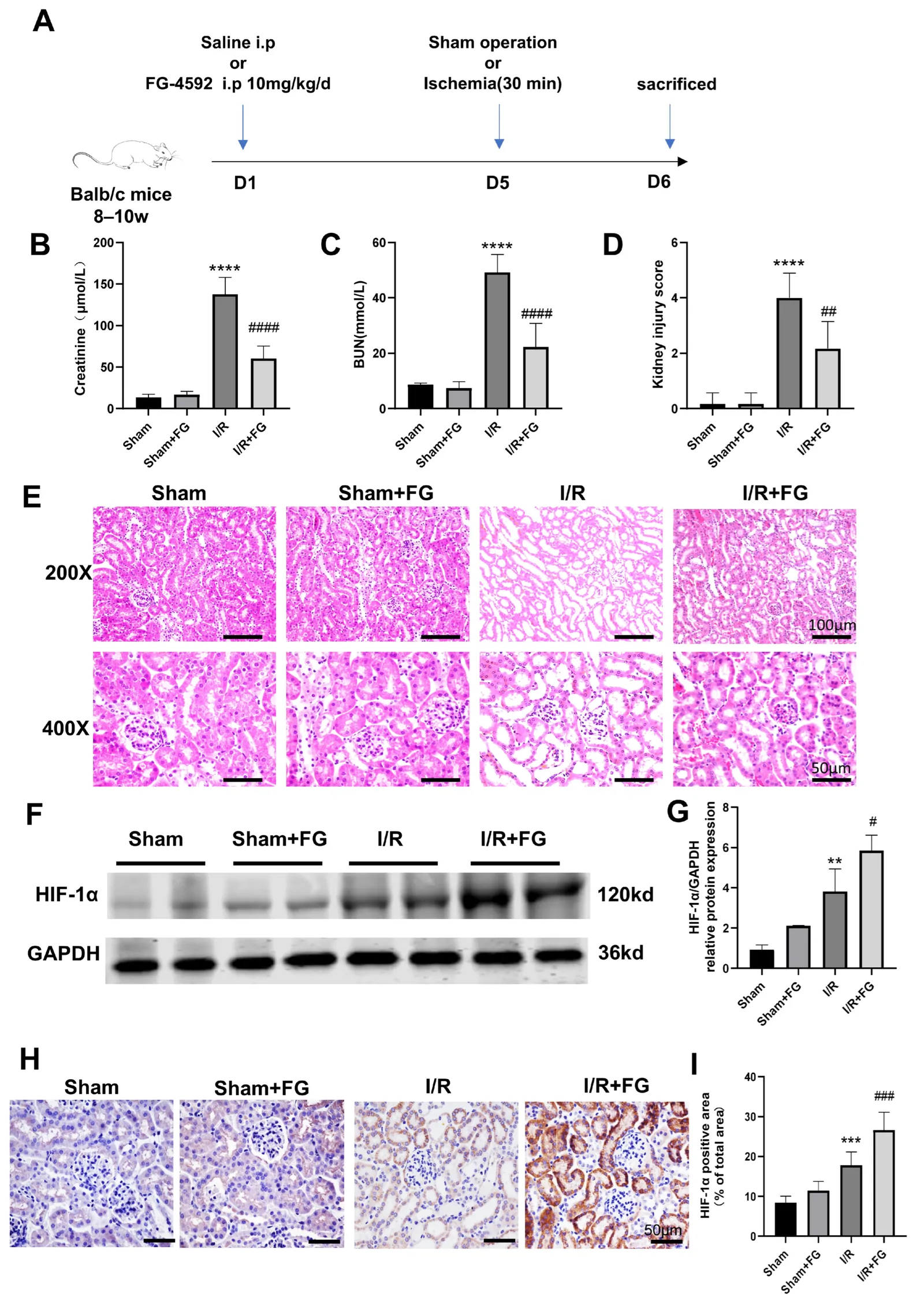
Impact of FG-4592 on renal injury and stabilization of HIF-1α in IRI mice model. (**A**) Experimental design timeline. Rodents were allocated into four groups (*n* = 6 per group): (1) Sham; (2) Sham + FG; (3) IR; (4) IR + FG. FG-4592 (10 mg/kg/day i.p.) was added for 5 days. (**B**) Serum creatinine. (**C**) Blood urea nitrogen. (**D**,**E**) Representative renal histology by H&E staining (×200, bar = 100 μm; ×400, bar = 50 μm) and analysis of tubular injury score. (**F**,**G**) Western blotting analysis and quantification of HIF-1α. GAPDH served as loading control. Densitometric quantification was based on biological replicates (*n* = 3). Representative blots show two samples per group. (**H**,**I**) Representative immunohistochemical images (×400, bar = 50 μm) and quantification of HIF-1α staining in renal cortical regions. Data are expressed as mean ± SEM (*n* = 6). ** *p* < 0.01, *** *p* < 0.001, **** *p* < 0.0001 versus Sham group; # *p* < 0.05, ## *p* < 0.01, ### *p* < 0.001, #### *p* < 0.0001 versus IR group. Original images of Figure 2F can be found in Figure S1.

Having established that MICA is elevated in human AKI/PRT biopsies, we next asked whether pharmacological HIF stabilization with FG-4592 would protect against renal IRI and regulate the murine NKG2D ligands RAE-1 and H60 in vivo. To investigate the impact of HIF stabilization on renal IRI, we subjected mice to a unilateral IR model with or without pretreatment with FG-4592. The experimental protocol is illustrated in Figure 2A. Pretreating mice with FG-4592 improved kidney function, indicated by reduced SCr and BUN versus saline controls (Figure 2B,C; SCr: Sham, 13.67 ± 1.48 μmol/L; Sham + FG, 16.83 ± 1.58 μmol/L; IR, 137.70 ± 8.24 μmol/L; IR + FG, 60.33 ± 6.19 μmol/L; overall ANOVA *p* < 0.0001; IR vs. IR + FG, *p* < 0.0001. BUN: Sham, 8.64 ± 0.23 mmol/L; Sham + FG, 7.40 ± 0.95 mmol/L; IR, 49.22 ± 2.65 mmol/L; IR + FG, 22.34 ± 3.47 mmol/L; overall ANOVA *p* < 0.0001; IR vs. IR + FG, *p* < 0.0001). Histologically, it alleviated the substantial tubular injury observed in controls 24 h post-IR (Figure 2D,E) (tubular injury score: Sham, 0.17 ± 0.17; Sham + FG, 0.17 ± 0.17; IR, 4.00 ± 0.37; IR + FG, 2.17 ± 0.40; overall ANOVA *p* < 0.0001; IR vs. IR + FG, *p* = 0.0003). Renal IRI promoted HIF-1α expression, with FG-4592 treatment further elevating its protein levels, as confirmed by Western blot analysis (Figure 2F,G; HIF-1α/GAPDH relative protein expression: Sham, 0.93 ± 0.13; Sham + FG, 2.12 ± 0.01; IR, 3.82 ± 0.64; IR + FG, 5.85 ± 0.44; overall ANOVA *p* = 0.0001; IR vs. IR + FG, *p* = 0.0067) and IHC (Figure 2H,I; HIF-1α-positive area: Sham, 8.43 ± 0.65% of total area; Sham + FG, 11.47 ± 0.94%; IR, 17.82 ± 1.36%; IR + FG, 26.66 ± 1.81%; overall ANOVA *p* < 0.0001; IR vs. IR + FG, *p* < 0.0001). As expected based on the pharmacological mechanism of FG-4592 as a PHD inhibitor, FG-4592 treatment in sham-operated mice also increased HIF-1α protein levels compared to vehicle-treated shams, reflecting HIF-1α stabilization even under normoxic conditions. The IR + FG group showed the highest HIF-1α levels, indicating an additive effect of ischemic stress and pharmacological PHD inhibition. Collectively, these findings revealed that FG-4592 pretreatment conferred nephroprotection and promoted HIF-1α stabilization in mice with renal IR injury.

### 3.3. FG-4592 Induced the Expression of NKG2D Ligands RAE-1 and H60 in IR-AKI Murine Model

**Figure 3:**
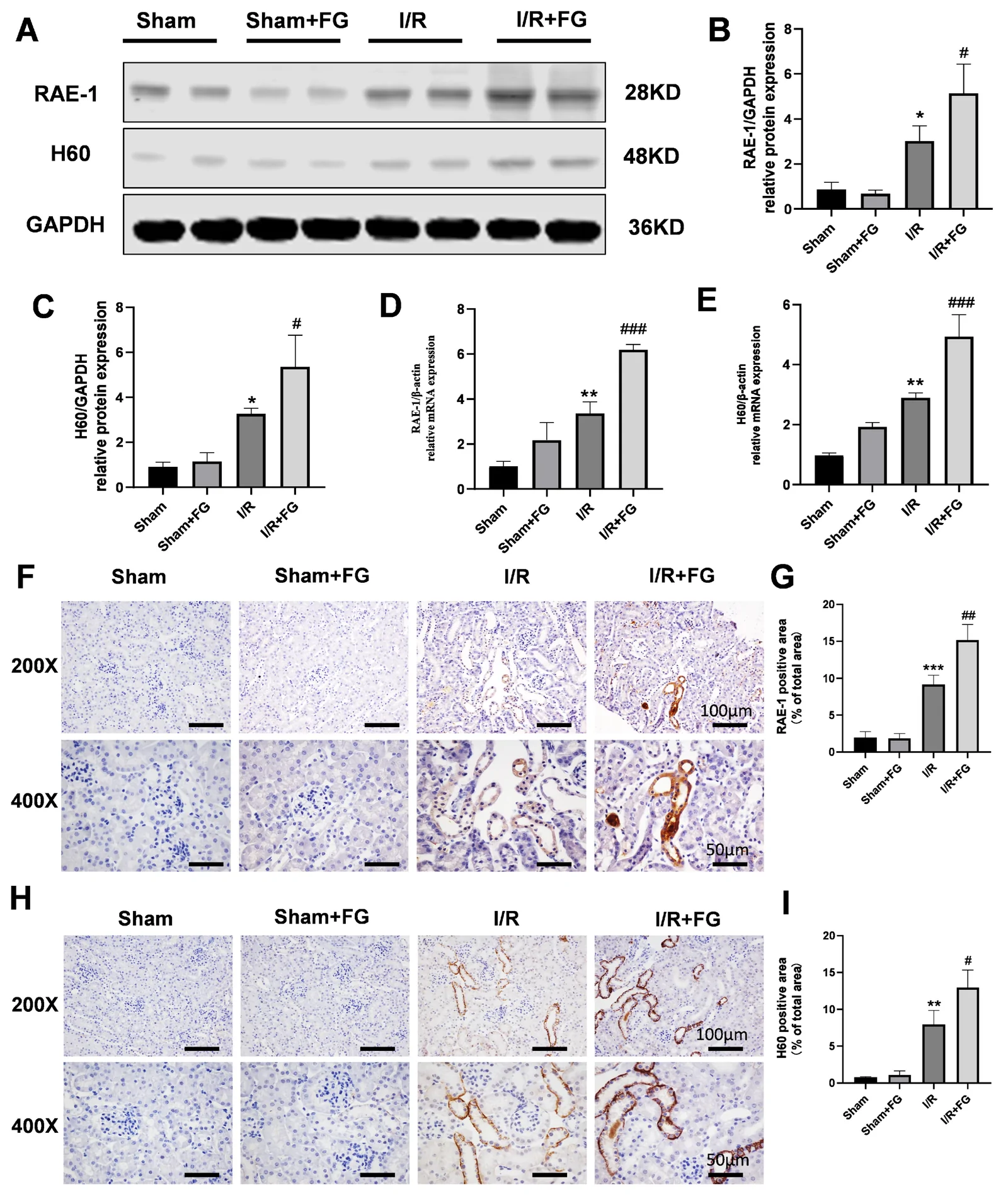
FG-4592 enhances NKG2D ligand expression in IR-AKI murine model. (**A**–**C**) Western blot analysis and quantification of RAE-1/H60, with GAPDH serving as loading control. Densitometric quantification was based on biological replicates (*n* = 3). Representative blots show two samples per group. (**D**,**E**) Quantitative PCR analysis of renal RAE-1 and H60 mRNA levels in sham-operated, IR with saline treatment, and IR with FG-4592 treatment groups (*n* = 3 randomly selected from the 6 mice per group; each sample run in triplicate). (**F**–**I**) Representative immunohistochemical images (×200, bar = 100 μm; ×400, bar = 50 μm) and quantification of RAE-1 and H60 staining in renal cortical region. Data are expressed as mean ± SEM, * *p* < 0.05, ** *p* < 0.01, *** *p* < 0.001 versus Sham group; # *p* < 0.05, ## *p* < 0.01, ### *p* < 0.001 versus IR group. Original images of Figure 3A can be found in Figure S2.

We next assessed whether FG-4592 regulates NKG2D ligands in renal IRI. Western blot analysis showed that ischemic injury upregulated RAE-1 and H60 in mouse kidneys, an effect further augmented by FG-4592 treatment compared to saline controls (Figure 3A–C; RAE-1/GAPDH relative protein expression: Sham, 0.87 ± 0.18; Sham + FG, 0.68 ± 0.10; IR, 3.02 ± 0.39; IR + FG, 5.14 ± 0.75; overall ANOVA *p* = 0.0003; IR vs. IR + FG, *p* = 0.0088. H60/GAPDH relative protein expression: Sham, 0.91 ± 0.12; Sham + FG, 1.16 ± 0.22; IR, 3.27 ± 0.14; IR + FG, 5.36 ± 0.81; overall ANOVA *p* = 0.0003; IR vs. IR + FG, *p* = 0.0087). At the transcriptional level, RT-PCR further confirmed this upregulation, revealing elevated mRNA levels of both ligands (Figure 3D,E; RAE-1/β-actin relative mRNA expression: Sham, 1.01 ± 0.12; Sham + FG, 2.18 ± 0.45; IR, 3.36 ± 0.29; IR + FG, 6.19 ± 0.14; overall ANOVA *p* < 0.0001; IR vs. IR + FG, *p* = 0.0001. H60/β-actin relative mRNA expression: Sham, 0.97 ± 0.04; Sham + FG, 1.93 ± 0.08; IR, 2.90 ± 0.09; IR + FG, 4.94 ± 0.42; overall ANOVA *p* < 0.0001; IR vs. IR + FG, *p* = 0.0002). Accordingly, immunohistochemical analysis demonstrated that IR injury increased the staining area for NKG2D ligands compared to the sham group, and this increase was more pronounced with FG-4592 treatment (Figure 3F–I; RAE-1-positive area: Sham, 1.97 ± 0.47% of total area; Sham + FG, 1.86 ± 0.38%; IR, 9.20 ± 0.71%; IR + FG, 15.17 ± 1.21%; overall ANOVA *p* < 0.0001; IR vs. IR + FG, *p* = 0.0006. H60-positive area: Sham, 0.77 ± 0.06%; Sham + FG, 1.09 ± 0.30%; IR, 7.96 ± 1.10%; IR + FG, 12.95 ± 1.38%; overall ANOVA *p* < 0.0001; IR vs. IR + FG, *p* = 0.0043). Together, these data indicated that FG-4592 enhances the upregulation of NKG2D ligands triggered by IR injury in mouse kidneys.

### 3.4. FG-4592 Enhanced NKG2D Ligand Expression in Hypoxic–Reoxygenated HK-2 Cells and mTECs

**Figure 4:**
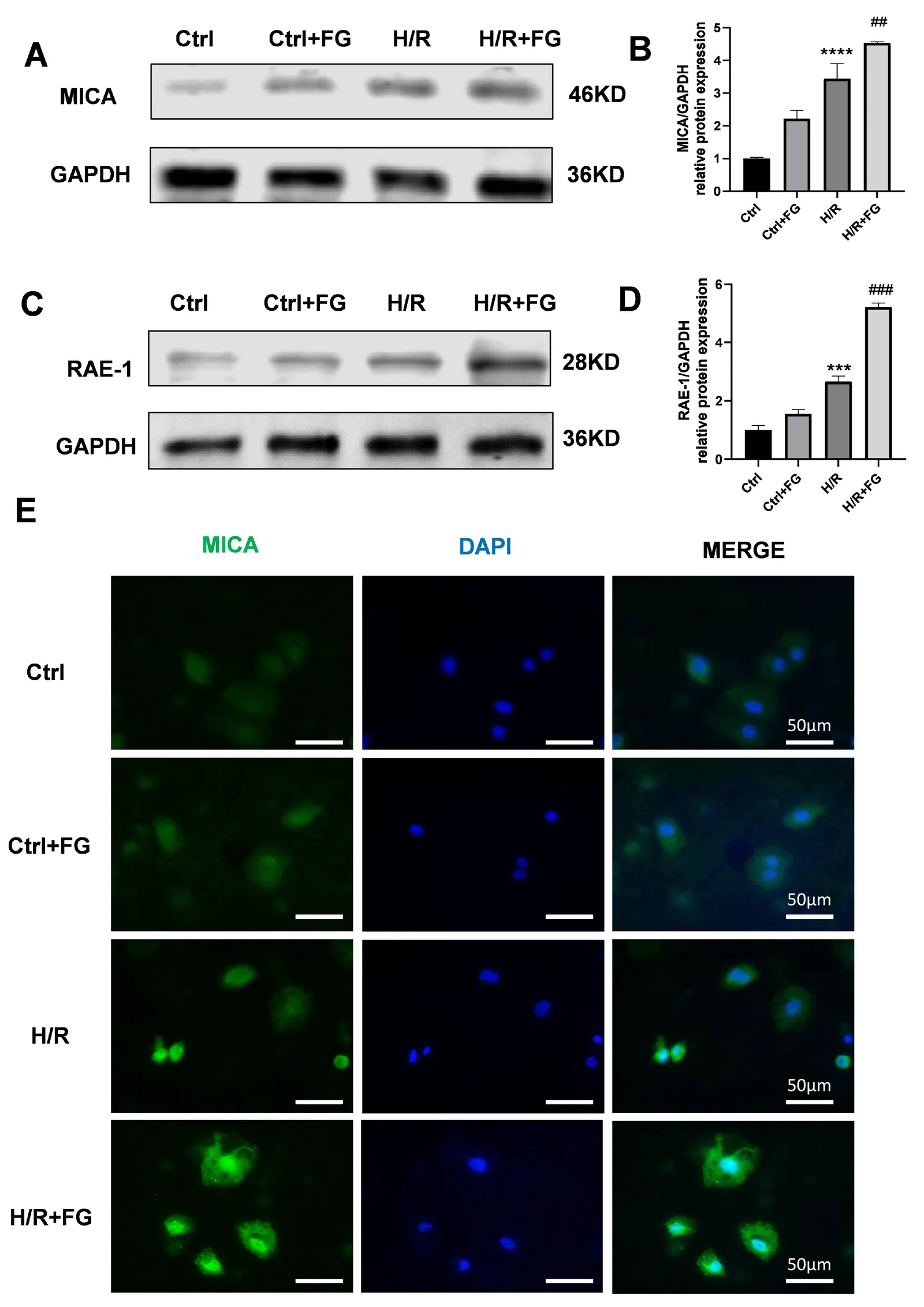
FG-4592 enhances human MICA and mouse RAE-1 expression under hypoxia/reoxygenation in vitro. For FG-4592 pretreatment, cells were incubated for 24 h before hypoxia induction. The cellular models underwent 24 h of hypoxia followed by 2 h of reperfusion. Normoxic cultured HK-2 cells were utilized as controls. (**A**,**B**) Western blot assays were conducted to assess the human MICA expression in HK-2 cells. Densitometric quantification was based on biological replicates (*n* = 3). (**C**,**D**) Western blot assays measured RAE-1 expression patterns in mTEC cells. Densitometric quantification was based on biological replicates (*n* = 3). (**E**) Representative immunofluorescent images (×400, bar = 50 μm) illustrate MICA distribution in HK-2 cells, displaying separate panels for MICA (green), DAPI (blue), and the merged view. Representative images are from one independent experiment; similar results were observed in at least three independent experiments. Data are expressed as mean ± SEM, *** *p* < 0.001, **** *p* < 0.0001 versus the control group; ## *p* < 0.01, ### *p* < 0.001 versus H/R group. Original images of Figure 4A,C can be found in Figure S3.

To determine whether the FG-4592-mediated enhancement of NKG2D ligands reflects a direct effect on renal tubular epithelial cells, independent of systemic immune influences, we next performed hypoxia/reoxygenation experiments in human HK-2 cells and murine mTECs. To specifically investigate the involvement of NKG2D ligands in renal H/R injury and the regulatory effect of FG-4592 on these ligands, we examined their expression in in vitro H/R models. Our data show that in HK-2 cells, H/R increased expression of the human NKG2D ligand MICA, and FG-4592 enhanced this increase (Figure 4A,B; MICA/GAPDH relative protein expression: Ctrl, 1.00 ± 0.09; Ctrl + FG, 1.55 ± 0.09; H/R, 2.66 ± 0.11; H/R + FG, 5.21 ± 0.08; overall ANOVA *p* < 0.0001; H/R vs. H/R + FG, *p* < 0.0001). A similar trend was observed for RAE-1 in mTECs (Figure 4C,D; RAE-1/GAPDH relative protein expression: Ctrl, 1.00 ± 0.03; Ctrl + FG, 1.25 ± 0.07; H/R, 4.31 ± 0.58; H/R + FG, 9.07 ± 0.56; overall ANOVA *p* < 0.0001; H/R vs. H/R + FG, *p* < 0.0001). Immunofluorescence analysis corroborated these findings, clearly visualizing the FG-4592-mediated boost in MICA protein levels under H/R (Figure 4E). Thus, FG-4592 potentiates the H/R-triggered upregulation of NKG2D ligands across species. Together, these cross-species in vitro findings provide mechanistic support for the in vivo results.

## 4. Discussion

Evidence suggests that renal hypoxia and associated immune response significantly contribute to the development of IRI-induced AKI [26,27]. Cellular adaptation in IRI is largely regulated by the transcription factor HIF [28]. Meanwhile, IRI is a crucial stimulator for upregulating many stress molecules, including NKG2D ligands [9]. Identifying these stress-related events in IRI is vital for understanding their impact on renal prognosis and formulating prevention strategies. Together, we characterized a novel regulatory pathway linking pharmacological HIF stabilization to NKG2D ligand expression in IRI-induced AKI. For the first time, our data establish that MICA protein levels are significantly elevated in the tubular epithelium of patients with either AKI or PRT patients with renal dysfunction. We also confirmed the upregulation of murine NKG2D ligands RAE-1 and H60 in renal IRI, both in vivo and in vitro. Roxadustat (FG-4592) pretreatment exerted a nephroprotective effect in renal IRI, as previously reported. However, this HIF-PHI unexpectedly induced the upregulation, not downregulation, of NKG2D ligands in renal tubular epithelial cells, although these ligands are conventionally viewed as immune-promoting. This paradoxical effect, in which a protective agent increases putative pro-inflammatory signals, has not been reported before and suggests a novel immunomodulatory axis.

MICA, a member of the NKG2D ligand family, shares structural homology with MHC class I molecules. Its binding to NKG2D on immune cells overrides inhibitory signals, thereby promoting cytotoxicity and inflammation. MICA has gained clinical attention in renal transplantation, where it functions as an independent transplantation antigen that directly influences allograft survival [5,6,29]. We detected upregulated MICA in renal tubular epithelial cells during ATN in AKI and PRT patients with renal insufficiency, an effect not previously reported. As described in our prior research [18], we deliberately excluded individuals with immunological or nephrotoxic etiologies to ensure renal injury was due to IRI as much as possible. Of note, the human biopsies were obtained from patients who did not receive Roxadustat. Thus, while these data support the clinical relevance of MICA upregulation in human IRI-associated ATN, the pharmacological effect of FG-4592 on NKG2D ligands is demonstrated in our murine IRI model and in vitro H/R experiments. Our study enrolled a relatively small number of patients. Thus, this result should be validated in a larger cohort. Since this was a retrospective biopsy-registry study, some more granular clinical details—such as the exact timing of AKI onset and specific precipitating factors—were not uniformly recorded. We therefore report only the information that could be reliably obtained, and we acknowledge this as a limitation of the present study. Nevertheless, all patients had biopsy-proven ATN, and the available clinical data were sufficient to support the main findings of this study. Our current investigation demonstrated increased levels of mouse NKG2D ligands in both in vitro and in vivo settings of IR-induced kidney injury. Although phylogenetically distinct from human MICA, murine NKG2D ligands (including RAE-1/H60 families and MULT1) are functionally analogous in stress-inducible expression and immune activation capacity. This functional conservation validates murine models for NKG2D pathway research [30,31]. We also observed MICA upregulation in HK-2 cells subjected to H/R conditions, indicating MICA involvement in human renal IRI. Feng et al. [11] found that IRI elevates RAE-1 expression in C57BL/6 mouse kidneys, implying that NKG2D ligands might serve as important mediators connecting innate and adaptive immune responses in ischemic injury. Zhang et al. reported that the NKG2D/RAE-1 interaction promotes renal IRI by mediating direct, perforin-dependent NK cell killing of murine TECs, a finding supported by attenuated functional impairment after NK cell blockade [7]. This indicates the detrimental role of NKG2D activation in IRI. The pathogenic role of the NKG2D axis extends to lung IRI [12] and myocardial infarction, in which its inhibition attenuates remodeling [13]. Together, these data identify NKG2D ligands as key factors in renal ischemic injury. Defining their expression patterns and roles may thus inform innovative therapeutic strategies for renal IRI.

Our study demonstrates that Roxadustat (FG-4592) exerts a nephroprotective effect in both IRI mouse and H/R cell models, lowering SCr/BUN levels and reducing morphological damage. Given the central role of HIFs in regulating gene expression during hypoxic injury and repair [14], stabilizing HIF has become a promising therapeutic strategy for IRI. Consistent with our findings, several preclinical studies have demonstrated the nephroprotective effects of the clinically widely used HIF-PHI Roxadustat (FG-4592) in IRI models, beyond its use for anemia. We previously reported that FG-4592 mitigates IRI-induced AKI via anti-inflammatory mechanisms, including enhanced CD73 and suppressed AIM2 inflammasomes [18]. Similarly, Miao et al. found that FG-4592 ameliorated kidney injury in a murine IRI model, reducing immune cell infiltration and inflammatory cytokines [20]. A study by Zhang et al. revealed that FG-4592 may ameliorate IRI-induced AKI through suppression of HIF-1α-associated mitophagy in renal tubular cells [21]. Another novel HIF-PHI, Enarodustat, exerts ischemic protection via HIF-1-driven upregulation of glycogen synthesis-related genes, thereby increasing renal glycogen storage [19]. Collectively, HIF-PHIs are reported to activate cytoprotective programs under hypoxia, regulating genes involved in inflammation suppression, metabolic reprogramming, and apoptosis inhibition, thereby mitigating IRI. Since MICA is also induced by IRI, we hypothesize that FG-4592 protects against IRI via the NKG2D pathway.

Surprisingly, our research on FG-4592’s impact on MICA revealed unexpected findings. Data from both in vivo and in vitro mouse models, along with human cell cultures, showed that this compound enhances NKG2D ligand expression, contradicting our initial hypothesis. This phenomenon has not been previously reported. In in vivo mouse models, while HIF-1α was detected in both proximal and distal tubules (Figure 2H), RAE-1 and H60 staining was predominantly observed in tubular structures that appeared to be distal tubules and collecting ducts (Figure 3F–I). Previous studies have reported NKG2D ligand expression in renal tubular epithelial cells without specifying proximal or distal localization [7,11,31]. In addition, the expression levels of NKG2D ligands can vary depending on the time point [7,11]. These observations suggest that the distribution of NKG2D ligands may be influenced by cell-type-specific regulatory mechanisms and the local microenvironment, rather than simply reflecting HIF-1α availability. Importantly, our in vitro experiments in proximal tubular cell lines (HK-2 and mTECs) confirmed that FG-4592 directly upregulates NKG2D ligands under H/R conditions.

The mechanism by which FG-4592 upregulates NKG2D ligands remains elusive. We propose two non-exclusive hypotheses. First, HIF-1α may directly transactivate ligand genes via promoter HREs, a possibility that warrants further investigation using methods such as chromatin immunoprecipitation. In support of this direct pathway, a prior study reported that HIF-1α overexpression via an adenoviral vector encoding oxygen-dependent degradation domain-deleted HIF-1α (HIF-1αΔODD) upregulates MICA in HK-2 cells subjected to hypoxia/reoxygenation conditions [32]. However, these data derive from overexpression systems without HIF-PHI treatment, leaving the direct relevance to FG-4592 uncertain. Second, HIF stabilization may indirectly induce these ligands through secondary mediators, such as inflammatory cytokines or metabolic stress signals. Given these uncertainties, additional in vivo or mechanistic research is needed to clarify the specific mechanisms underlying HIF-PHI-triggered elevation of NKG2D ligands.

NKG2D ligand upregulation aggravates IRI-associated organ injury, yet FG-4592 confers short-term renoprotection in the same model. Several non-exclusive explanations may reconcile this paradox. One possibility is that FG-4592 confers acute protection primarily through mechanisms that override any NKG2D-driven damage, including anti-inflammatory effects, metabolic reprogramming, and improved mitochondrial function [19,20,33,34,35]. The second possibility is that the functional outcome of NKG2D ligand upregulation is not unidirectional. For instance, the soluble form (sMICA) generated by cleavage of membrane-bound MICA can suppress NKG2D-mediated responses. Oncology studies have demonstrated that sMICA, which is detectable in the circulation, binds to NKG2D on NK cells without triggering downstream signaling. Instead, sMICA competitively inhibits membrane MICA engagement, induces NKG2D internalization and degradation, and consequently impairs NK cell cytotoxicity, thereby facilitating tumor immune evasion [36]. HIF-1α, which is highly expressed in hypoxic tumor microenvironments, transcriptionally upregulates metalloproteinases such as MMP2, MMP9, and ADAM10. These enzymes cleave membrane MICA to generate sMICA, thereby driving the above immune escape pathway [37]. Thus, if this sMICA scenario applies to renal IRI, FG-4592 may similarly promote MICA shedding through HIF-1α stabilization, thereby exerting an immunosuppressive effect, although this possibility awaits clinical validation. Of note, this hypothetical shedding does not necessarily conflict with our observation of increased membrane MICA, as the two processes may coexist—upregulated membrane MICA serves as the substrate for subsequent proteolytic cleavage. Because mice lack the MICA gene, serum sMICA could not be measured in our model. Nevertheless, this limitation does not undermine our primary finding that FG-4592 upregulates membrane NKG2D ligand (MICA) expression. The third possibility is the effect of downstream mediators of FG-4592. FG-4592 is known to induce EPO expression through HIF stabilization. EPO exerts tissue-protective and immunomodulatory effects, including inhibition of pro-inflammatory cytokines [38], promotion of macrophage M2 polarization [39,40], and induction of regulatory T cells [41]. However, whether EPO directly modulates NKG2D ligand expression or NKG2D signaling remains unknown. Given that the immunomodulatory actions of EPO are generally anti-inflammatory, they may operate through pathways independent of NKG2D ligand regulation, or they might partially counterbalance the pro-inflammatory potential of NKG2D ligand upregulation. Future studies using human renal tubular epithelial cells or clinical samples from patients receiving HIF-PHIs are warranted to determine whether FG-4592 modulates MICA in humans and to explore the potential interplay between EPO and NKG2D signaling in renal IRI. Despite the short-term renoprotection observed and the potential mechanisms discussed above, our findings nevertheless raise a critical concern that prolonged FG-4592 treatment with sustained NKG2D ligand elevation might predispose the patient to long-term immune injury. Therefore, longitudinal follow-up studies are needed to evaluate the safety of extended FG-4592 treatment.

## 5. Conclusions

In summary, our findings identify Roxadustat as nephroprotective in IRI-AKI. Despite its upregulation of NKG2D ligands in ischemic kidneys, this change did not worsen renal injury during the acute phase. The long-term clinical consequences of HIF-PHI–driven ligand induction, particularly in AKI and PRT patients, warrant investigation. Prospective studies should also clarify whether and how Roxadustat, or HIF-1α and its downstream mediators, alter membrane-bound and soluble MICA in patients with renal IRI.

## Figures and Tables

**Table 1:** Sequences of the primers for quantitative real-time PCR.

Gene	Forward Primer (5′-3′)	Reverse Primer (3′-5′)
Mouse RAE-1	ACCATGATTTATCCGCAAAGCC	CTGATCTCCAGCTCATATTCTCTGTG
Mouse H60	ACAGTGTCACCTCACACTGTTCAG	TTATTTTTCTAACCCACCGCTCC
Mouse β-actin	CATTGCTGACAGGATGCAGAAGG	TGCTGGAAGGTGGACAGTGAGG

**Table 2:** General information of AKI patients, PRT patients, and normal controls.

Patients	Gender	Age (Years)	SCr (μmol/L)	BUN (mmol/L)	Cystatin C (mg/L)	eGFR (ml/min/1.73 m^2^)	Albumin (g/L)	UTP (g/24 h)	Diagnosis
1	M	15	174	11.0	2.6	49.13	40	2.15	AKI S2; ATN
2	F	73	285	25.2	3.32	13.6	32	0.54	AKI S2; ATN
3	F	53	235	9.8	2.92	19.71	46	0.78	AKI S2; ATN
4	F	29	327	18.1	3.37	15.65	29	1.4	AKI S3; ATN
5	M	20	737	13.4	2.97	7.8	42	0.27	AKI S3; ATN
6	M	35	177	11	1.89	41.9	47	0.08	AKI S2; ATN
7	M	56	369	18.1	3.13	14.9	44	1.09	PRT; ATN
8	M	29	200	13.3	2.58	37.7	30	1.55	PRT; ATN
9	M	47	170	21.6	2.94	36.8	39	0.57	PRT; ATN
10	F	21	215	9.5	2.64	29.3	47	0.24	PRT; ATN
11	F	35	142	7.5	2.23	41.5	39	0.44	PRT; ATN
12	M	32	129	9.2	1.52	62.8	42	0.81	PRT; ATN
13	M	62	67	4.7	0.80	92.9	42		RC (control)
14	F	57	54	5.3	0.93	84.7	39		RC (control)
15	F	54	47	3.6	0.69	100.8	41		RC (control)
16	F	55	64	9.4	0.90	87	42		RC (control)
17	M	66	58	6.3	0.65	125	41		RC (control)
18	M	53	61	4.9	0.9	113.8	35		RC (control)

Abbreviations: AKI, acute kidney injury; S, KDIGO standard; PRT, post-renal transplantation; RC, renal carcinoma; SCr, serum creatinine; BUN, blood urea nitrogen; eGFR, estimated glomerular filtration rate; UTP, urine total protein; ATN, acute tubular necrosis; M, male; F, female.

## Data Availability

Data generated in this research can be obtained by contacting the corresponding authors upon request.

## Supplementary Materials

The following supporting information can be downloaded at: https://www.mdpi.com/article/10.3390/biom16101437/s1, Figure S1: Representative original images of Western blotting in Figure 2F; Figure S2: Representative original images of Western blotting in Figure 3A; Figure S3: Representative original images of Western blotting in Figure 4A,C.
