# Supplementary material for: Roxadustat (FG-4592) Attenuates Renal Ischemia–Reperfusion Injury but Upregulates the NKG2D Ligand MICA
Source: Biomolecules. 2026 Oct 2;16(10):1437. doi: 10.3390/biom16101437 (PMC13702372; doi:10.3390/biom16101437)

**Figure S1**. Representative original images of Western blotting in Figure 2F

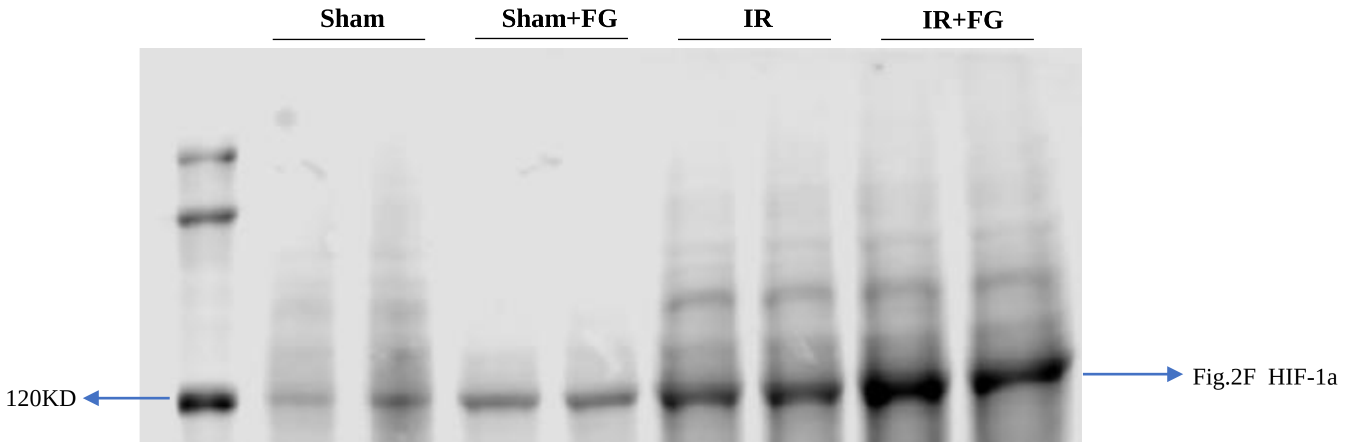


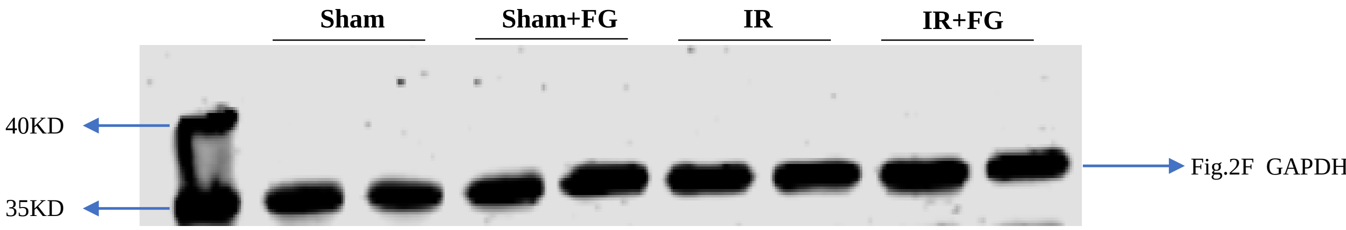

**Figure S2**. Representative original images of Western blotting in Figure 3A

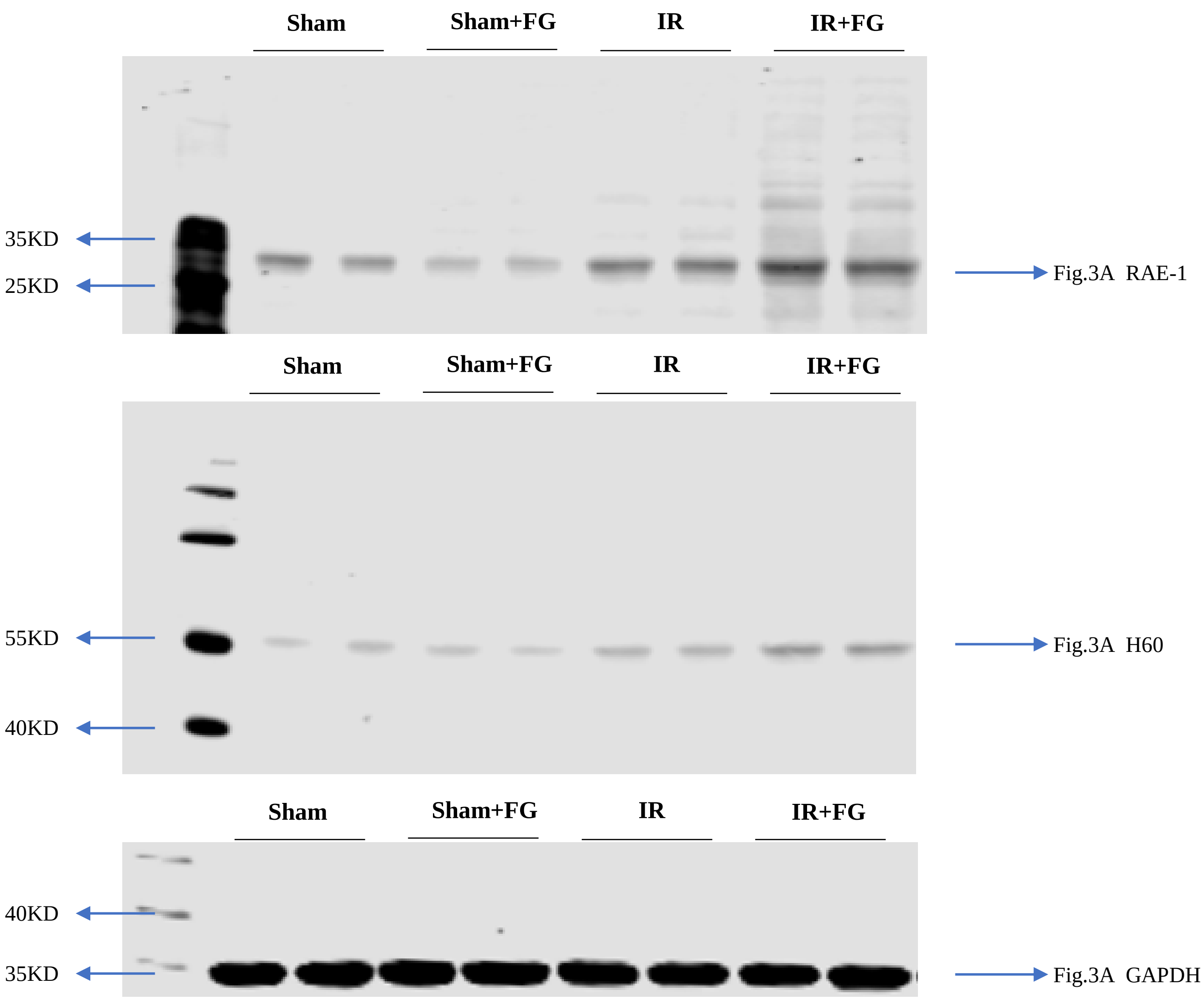

**Figure S3**. Representative original images of Western blotting in Figure 4A, C

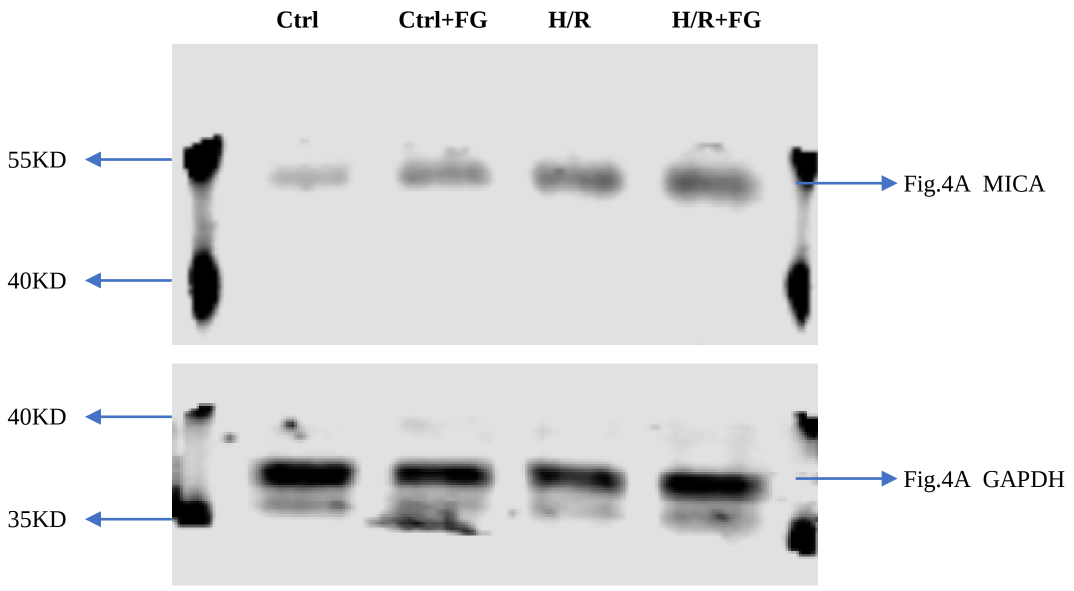


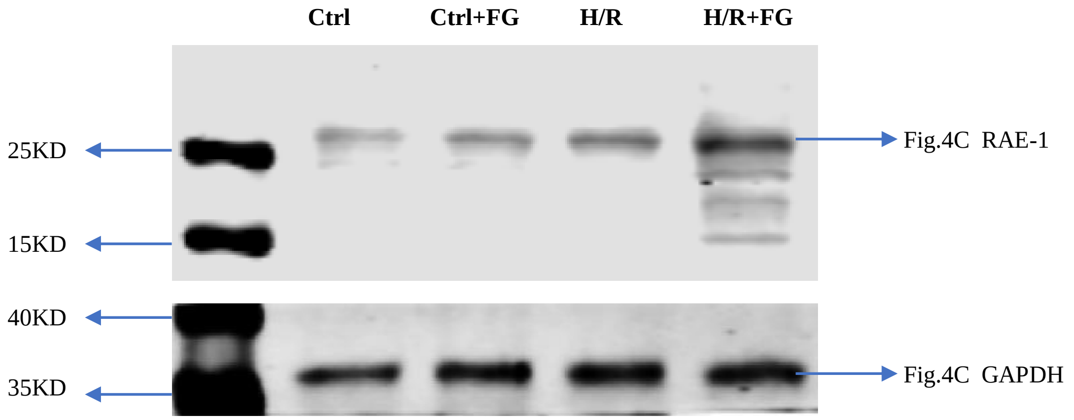

Supplement: Supplementary file 1 [file biomolecules-16-01437-s001.zip › biomolecules-4537290-supplementary.pdf]
